# Partial Calcanectomy in High-Risk Patients With Diabetes: Use and Utility of a “Hurricane” Incisional Approach

**Published:** 2010-02-01

**Authors:** Timothy K. Fisher, David G. Armstrong

**Affiliations:** Southern Arizona Limb Salvage Alliance, Department of Surgery, University of Arizona College of Medicine, 1501 N Campbell Ave, Tucson, AZ 85724.

## Abstract

**Introduction:** Plantar heel ulcers in people with diabetes represent a difficult challenge to the treating physician. They become even more difficult with underlying osteomyelitis. When this infection is in the calcaneus it typically results in a partial or total calcanectomy or even more frequently, high-level amputation. **Methods:** In this article, we describe a novel serpentine incisional approach to the plantar and (if necessary) posterior heel allowing for ample exposure and facilitating closure predominantly along relaxed skin tension lines. **Results:** We present several representative case examples in which a hurricane incision has been used to treat and provide closure to plantar-based calcaneal ulcers. **Discussion:** The use of this incision, which resembles a satellite view of a hurricane, was successful in achieving a desired partial calcanectomy and wound closure. This may be an additional tool in the armamentarium of the surgeon to assist in healing and amputation prevention.

People with diabetes have a 25% lifetime risk to develop a foot wound.^1^ Typically, more than half of these ulcers will become clinically infected.^2^,^3^ Lavery et al^4^ have recently shown that the risk factors for developing osteomyelitis in patients with diabetic foot wounds include deep wounds, penetrating to bone, a previous history of foot ulceration, or recurrent or multiple foot wounds. Plantar heel ulcers in people with diabetes represent a difficult challenge to the treating physician. They become even more difficult with underlying osteomyelitis. When this infection is in the calcaneus, it typically results in a partial or total calcanectomy or, even more frequently, high-level amputation.^5^

Partial calcanectomy was likely first described in the literature in 1931, when Gaenslen^6^ published his manuscript, describing splitting of the calcaneus into 2 halves, medial and lateral, and debriding all infected bone. Since that time, other authors such as Wilste et al,^7^ Crandall and Wagner,^8^ and Baravarian et al^9^ have all shown success with a partial calcanectomy.

Gaenslen's incision has typically been the approach of choice for a partial calcanectomy. Other incisions in the literature include a median longitudinal incision dividing the Achilles tendon, an incision beginning proximal to the insertion of the Achilles tendon and progressing distally to the calcaneocuboid joint, a horseshoe-shaped incision, a transverse incision superior to the calcaneus, a modified semielliptical longitudinal incision to remove a plantar ulcer.^10^

We report examples, one successful and one less-so, where a serpentine plantar-based incision was used to excise the ulceration, perform a partial calcanectomy, and close the wound. This approach is ideal to use in patients who suffer from a plantar heel ulcer when excessive surgical exposure to the calcaneus and posterior leg is to be avoided. It also reduces the size of resection of the posterior calcaneus when the area that is affected is primarily plantar. In both examples, the wounds were closed primarily given the absence of active infection and in order to eliminate the primary cause of the surgical intervention, the chronic ulcer. A plantar-based incision is made, with 2 semielliptical incisions circumscribing the calcaneal ulceration in order to resect the ulcer in total (Fig 1a). The incision is continued both proximally and distally from the apices of the resected wound. Full-thickness soft tissue dissection is performed to expose the calcaneus plantarly (Fig 1b). At this point, an osteotome or sagittal saw is used to resect the plantar prominence of the calcaneus or any devitalized bone. Once this has been completed, the skin is then reapproximated manually to evaluate if there is any tension prior to closure. If tension is appreciated, further undermining of the soft tissue is indicated. The area is then irrigated and closed in a deep to superficial fashion per the surgeon's choice. A drain is also recommended after closure to help reduce any potential hematoma formation. This procedure has been nicknamed the “Hurricane Incision” because of its resemblance to that recognizable meteorological feature.

## CASE 1

**Figure 2:**
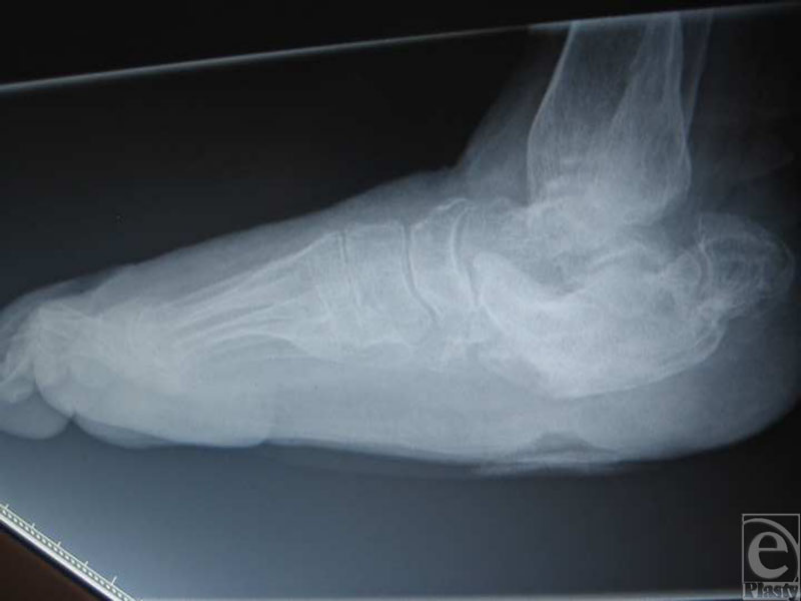

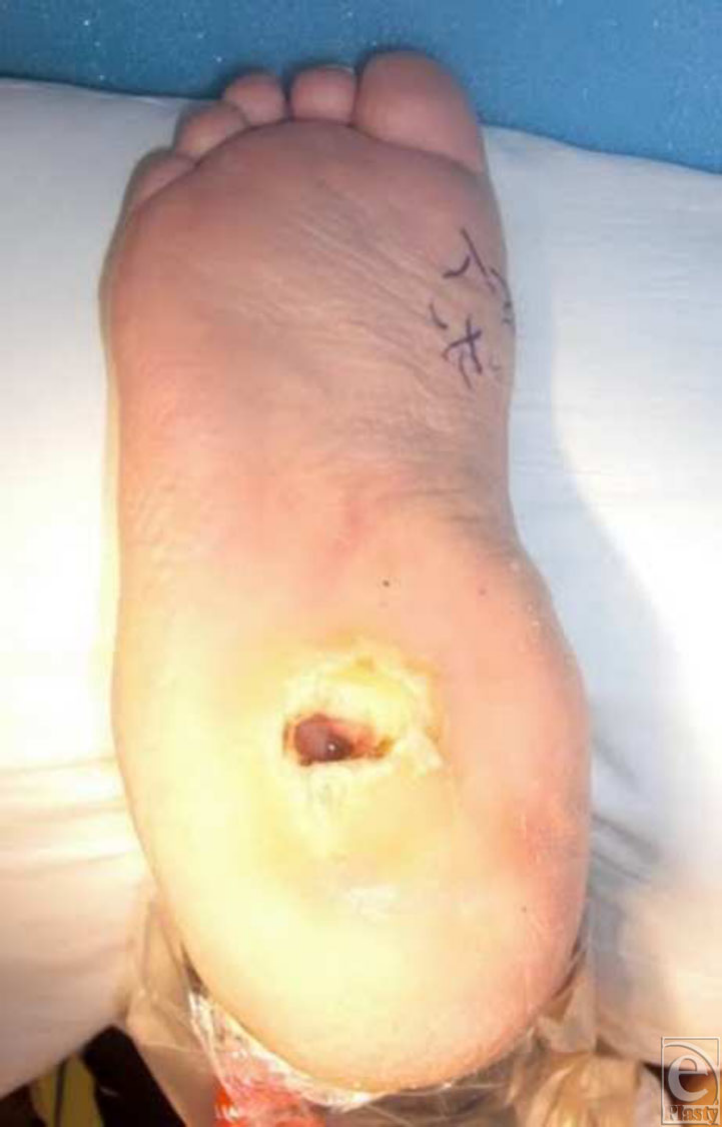

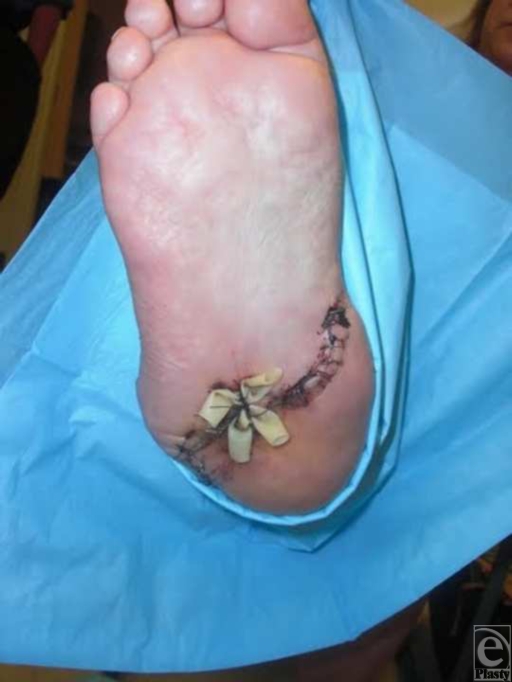

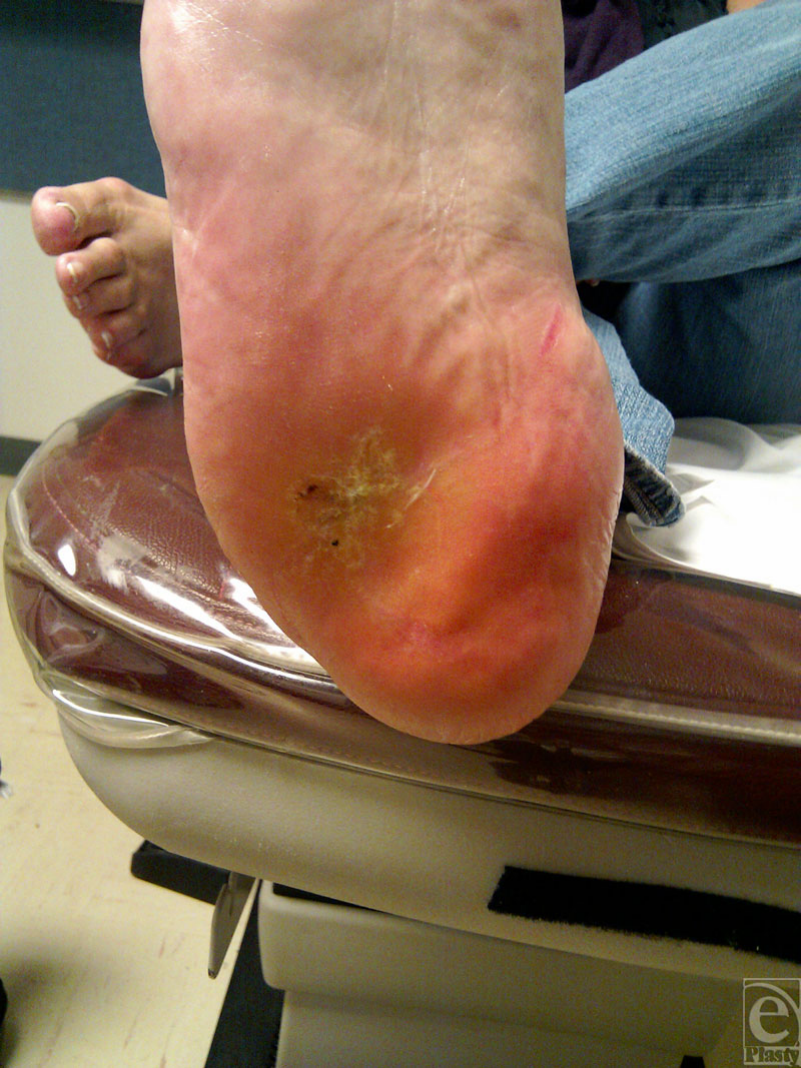
(a) Lateral radiograph showing plantarflexion of distal calcaneus causing the plantar ulceration. (b) Clinical picture of ulceration prior to surgical intervention. (c) First postoperative visit. (d) Wound healed at 6 weeks.

A 49-year-old woman with diabetes with a history of a plantar right-foot heel ulcer of 4-month duration initially presented to the SALSA clinics at the University of Arizona College of Medicine, Tucson. She had a history of Charcot changes to her midfoot and rearfoot making the calcaneus prominent to the plantar aspect (Fig 2a). She had undergone a previous surgical debridement of the ulcer, but it was unable to heal. After presenting to our clinic, she was managed with aggressive offloading, consisting of a CROW boot as well as standard ulcer care. With this combination, the ulcer became very superficial and was on the verge of being healed. She then went on vacation and returned with an increase in ulcer size and depth, probing the calcaneus (Fig 2b). To address this misadventure, surgical intervention was performed using the hurricane incision approach. The calcaneus was debrided and the incision was closed. Bolster sutures were used to the central aspect of the incision to reduce any potential tension. A Jackson-Pratt drain was placed, and the patient was placed in a posterior splint with strict non–weight-bearing orders. On her first postoperative visit (Fig 2c), the incision was well approximated with no signs of dehiscence or infection. The sutures were removed at 3 weeks.

## CASE 2

**Figure 3:**
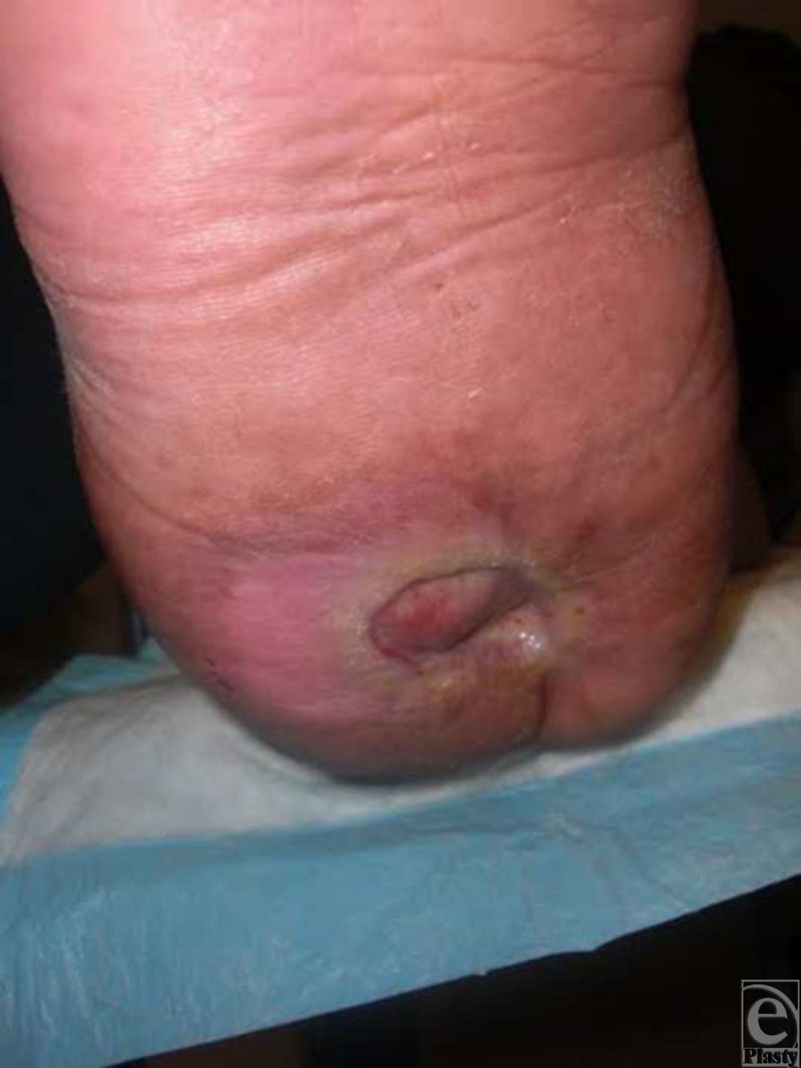

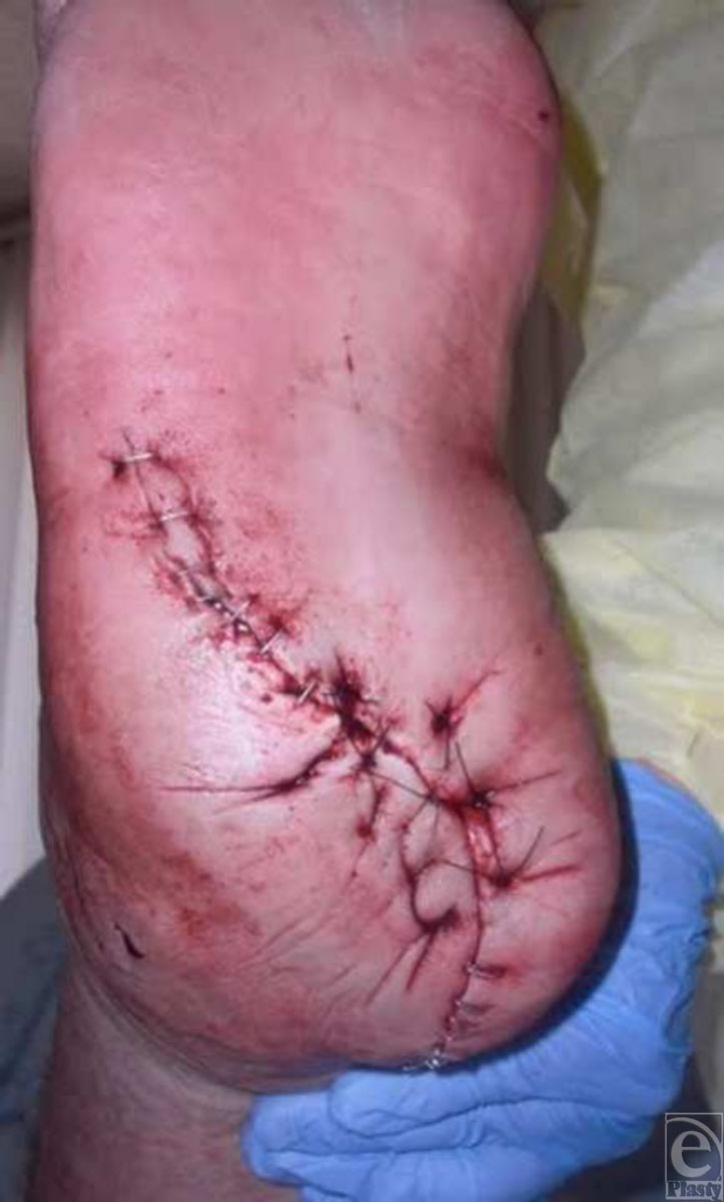

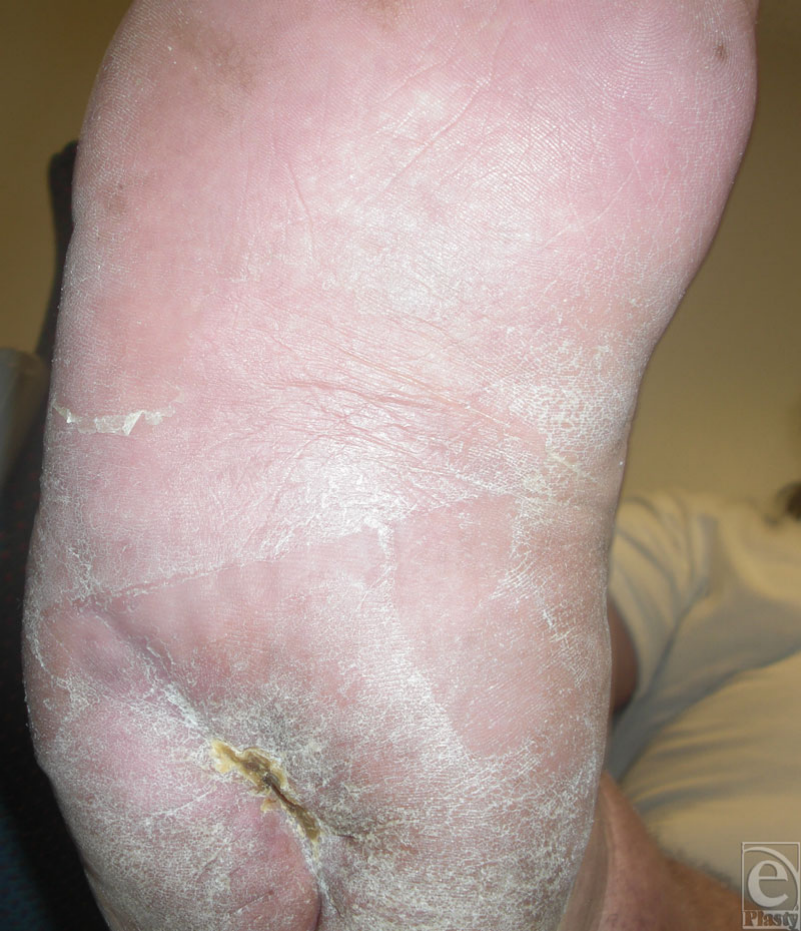
(a) Clinical picture of ulceration prior to surgical intervention. (b) Postoperative picture showing closure of ulceration. (c) Wound healed 2 months following partial calcanectomy with “hurricane” incision.

A 52-year-old gentleman with diabetes and morbid obesity with a chief complaint of a right heel ulcer of 5-months duration presented to the clinic. The ulcer formed as a result of a burn that he received while walking barefoot outside on a hot wooden deck. He had a history of a total right hip replacement in which nerve damage as a result of the surgery caused a right-foot drop and loss of sensation to the right plantar foot. After months of both surgical and conservative care, his ulcer was superficial but unable to close fully (Fig 3a). At this point, a partial calcanectomy with hurricane incisional approach was done to obtain ulcer closure. The calcaneus was debrided and the incision was closed (Fig 3b). At the second postoperative visit, the central aspect of the incision had dehisced, most likely because of pressure or weight bearing as the patient admitted to resting his foot on the ground. However, the wound was still far smaller than preoperatively and responded well to local wound care.

## CONCLUSION

Calcaneal ulcers present an interesting dilemma to the treating physician, especially when these ulcers become infected and develop underlying osteomyelitis. However, intervention in this case, whether it be partial or total calcanectomy, attempts to save limb length and can decrease the morbidity and mortality of these patients.^9^^-^11 One advantage, when using this approach, is that it may reduce the issue of what to do with the Achilles tendon. The Achilles may not be violated and can remain attached to its insertion in the calcaneus. Another advantage is that there is decreased exposure of the posterior aspect of a patient's leg and heel when the main issue is completely plantar. The plantar musculature and fascia are disrupted but, as reported, may be allowed to fibrose in situ postoperatively for a satisfactory result.^5^

Angiosomes are another important concept to keep in mind. They are defined as a 3-dimensional anatomic unit of tissue fed by a source artery.^12^ The plantar heel is supplied by the medial and lateral calcaneal arteries while the plantar midfoot is supplied mostly by perforators, on either side of the plantar fascia, from the medial and lateral plantar arteries. Attinger et al^13^ have suggested that either a coronal or sagittal incision on the heel is adequate for maintaining vascularity while on the plantar midfoot they advocate either a central midline incision or a Z-shaped incision in order to follow the angiosome boundary between the medial and lateral plantar arteries.^13^ The incision described in this article adheres to this concept and is versatile in that, when planning the proximal and distal wings, vascularity of the plantar heel and midfoot can be maintained.

Closure of the wounds primarily is advocated if there are no active signs of infection. This then eliminates the ulcer and any potential, continued, nidus for infection. The use of a hurricane-type incision for treatment of plantar heel ulcers, with or without underlying osteomyelitis, may yet be another tool in the armamentarium for the surgeon involved with diabetic limb salvage.

## Figures and Tables

**Figure 1:**
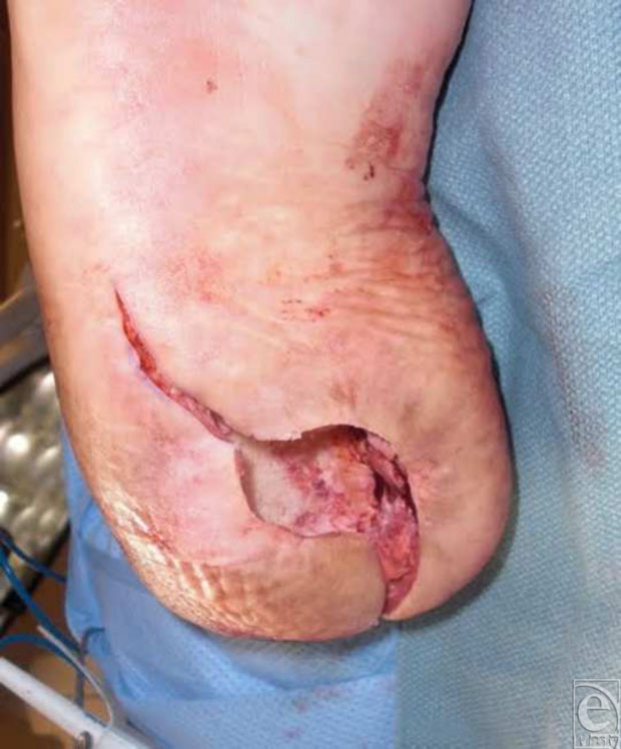

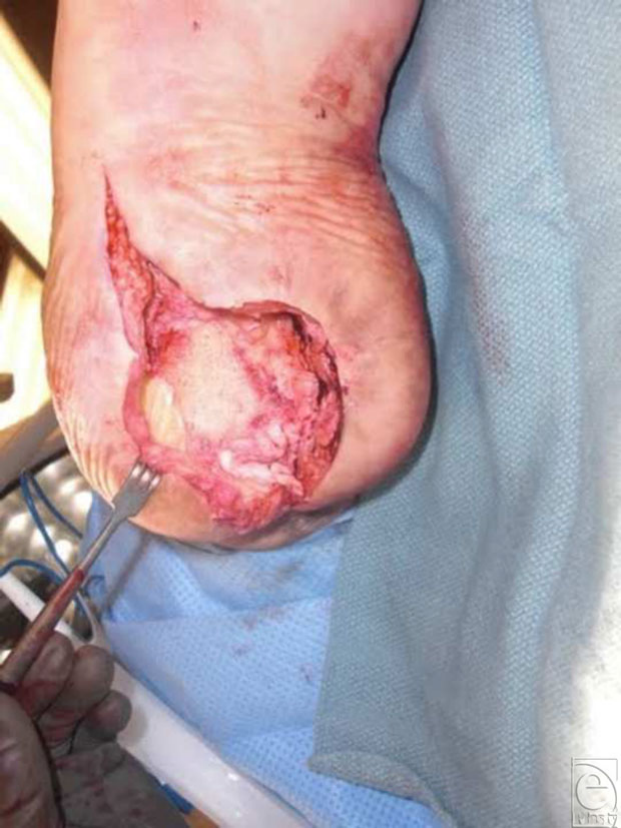
(a) Approach to Hurricane incision. (b) Exposure of the calcaneus after full-thickness dissection.
